# Supplementary material for: Black Ginseng Extract Exerts Potentially Anti-Asthmatic Activity by Inhibiting the Protein Kinase Cθ-Mediated IL-4/STAT6 Signaling Pathway
Source: Int J Mol Sci. 2023 Jul 26;24(15):11970. doi: 10.3390/ijms241511970 (PMC10418634; doi:10.3390/ijms241511970)
Supplement: Supplementary file 1 [file ijms-24-11970-s001.zip › ijms-2520355-supplementary.pdf]

Supplementary Information

Black Ginseng Extract Exerts Potentially Anti-Asthmatic Activity by Inhibiting the Protein Kinase Cθ-Mediated IL-4/STAT6 Signaling Pathway

Yu Na Song *et al.*,

● Supplementary Figures S1

Figure S1. Representative HPLC profiling of black ginseng extract (BGE) 1

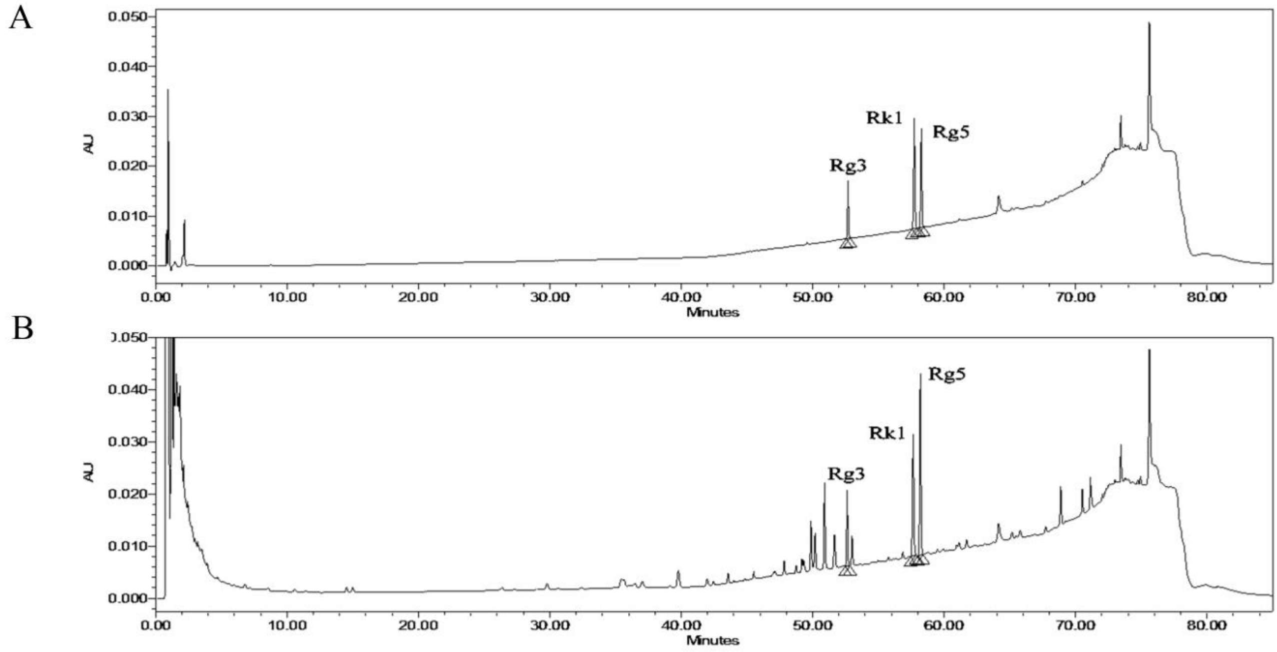

Figure S1. Representative HPLC profiling of black ginseng extract (BGE) (**A**–**B**) HPLC profiling chromatograms of three standard ginsenoside mixtures (**A**) and BGE (**B**) at λ = 203 nm. Rg3, Rk1, and Rg5 appeared at retention times of ~52.8, 57.9, and 58.2 min, respectively.
